# Nutritional Immunity Triggers the Modulation of Iron Metabolism Genes in the Sub-Antarctic Notothenioid *Eleginops maclovinus* in Response to *Piscirickettsia salmonis*

**DOI:** 10.3389/fimmu.2017.01153

**Published:** 2017-09-19

**Authors:** Danixa Martínez, Ricardo Oyarzún, Juan Pablo Pontigo, Alex Romero, Alejandro J. Yáñez, Luis Vargas-Chacoff

**Affiliations:** ^1^Instituto de Ciencias Marinas y Limnológicas, Universidad Austral de Chile, Valdivia, Chile; ^2^Escuela de Graduados, Programa de Doctorado en Ciencias de la Acuicultura, Universidad Austral de Chile, Puerto Montt, Chile; ^3^Centro Fondap de Investigación de Altas Latitudes (IDEAL), Universidad Austral de Chile, Valdivia, Chile; ^4^Centro Fondap Interdisciplinary Center for Aquaculture Research (INCAR), Universidad Austral de Chile, Valdivia, Chile; ^5^Instituto de Patología Animal, Universidad Austral de Chile, Valdivia, Chile; ^6^Instituto de Bioquímica y Microbiología, Universidad Austral de Chile, Valdivia, Chile

**Keywords:** *Eleginops maclovinus*, iron metabolism, notothenioid, nutritional immunity, iron-withholding

## Abstract

Iron deprivation is a nutritional immunity mechanism through which fish can limit the amount of iron available to invading bacteria. The aim of this study was to evaluate the modulation of iron metabolism genes in the liver and brain of sub-Antarctic notothenioid *Eleginops maclovinus* challenged with *Piscirickettsia salmonis*. The specimens were inoculated with two *P. salmonis* strains: LF-89 (ATCC^®^ VR-1361™) and Austral-005 (antibiotic resistant). Hepatic and brain samples were collected at intervals over a period of 35 days. Gene expression (by RT-qPCR) of proteins involved in iron storage, transport, and binding were statistically modulated in infected fish when compared with control counterparts. Specifically, the expression profiles of the transferrin and hemopexin genes in the liver, as well as the expression profiles of ferritin-M, ferritin-L, and transferrin in the brain, were similar for both experimental groups. Nevertheless, the remaining genes such as ferritin-H, ceruloplasmin, hepcidin, and haptoglobin presented tissue-specific expression profiles that varied in relation to the injected bacterial strain and sampling time-point. These results suggest that nutritional immunity could be an important immune defense mechanism for *E. maclovinus* against *P. salmonis* injection. This study provides relevant information for understanding iron metabolism of a sub-Antarctic notothenioid fish.

## Highlights

Iron deprivation is an innate immunity mechanism through which fish can limit the amount of iron available to invading bacteria.

The proteins involved in storage, transport, and iron binding modulated their expression during the experimental course.

The iron-related immune gene presented tissue-specific expression profiles that varied in relation to the injected bacterial strain (LF-89 or Austral-005) and sampling time-point.

## Introduction

The innate immune system in fish is an essential component in the fight against pathogenic agents. In contrast, the adaptive immune system is limited in fish due to being poikilothermic, having a restricted repertoire of antibodies, and presenting low lymphocyte proliferation, maturation, and memory (1). The cells and molecules of the innate immune system use non-clonal pattern recognition receptors, such as lectins, Toll-like receptors, and NOD-like receptors. These receptors not only recognize molecular structures essential for microorganism survival/pathogenicity (2) but also allow the innate immune response to rapidly respond to control pathogen growth and promote inflammation and adaptive immunity mechanisms (3).

Under inflammation and/or infestation conditions, the innate immune system induces various antimicrobial mechanisms, such as depleting the iron available to pathogens at the systemic and cellular levels (4). This defense mechanism, known as nutritional immunity or iron withholding, consists in the removal of this nutrient from circulation and posterior sequestration within cells (5). Proinflammatory cytokines, such as IL-6, stimulate the transcriptional upregulation of hepcidin, triggering and potentiating the hypoferric response of inflammation (6).

In eukaryotic cells and most prokaryotic organisms, iron is needed for survival and proliferation. This necessity arises as iron is a constituting element of hemoproteins, the iron-sulfur protein (Fe-S), and proteins that use iron in other functional groups to carry out essential functions of cellular metabolism (7). In fish, the iron preferentially crosses the apical membrane of gills and intestine in ferrous state (8) by the divalent metal transporter (9). Once inside the cell, iron can be stored in the cytoplasmic ferritin (i.e., iron that is not needed for immediate use) (10), sent toward the mitochondria (i.e., Fe-S cluster biogenesis or heme synthesis), or be used in other iron pathways (7).

Cellular iron export is regulated by hepcidin and ferroportin, the binding of both proteins leads the internalization and degradation of ferroportin (11). Ferroportin is responsible for iron transfer from the basolateral membrane of the cell to the blood (12), but iron export also depends on the presence of an associated copper, such as hephaestin (implicated in intestinal iron transport) and ceruloplasmin (implicated in the iron export from non-intestinal cells) (13, 14). This ferroxidase allows iron to be carried in the cytoplasmic transferrin and be sent to another tissue (7). Cellular iron uptake can occur through transferrin receptor 1 (TfR1), which is ubiquitously expressed in tissues (15), or through TfR2, a homologous receptor of TfR1 that is found in hepatic duodenal crypt cells and erythroid cells, localizations suggestive of a more specialized role for this receptor in iron metabolism (16).

When faced with an infectious process, the proteins involved in iron homeostasis can limit access to this nutrient. However, microorganisms have developed direct and indirect mechanisms for capturing iron from different sources *in vivo* (17). The direct capture of this element involves the expression of membrane proteins (i.e., receptors) that can directly bond with proteins that transport iron in the host. Iron can also be indirectly captured through the synthesis of hemophores and siderophores, which scavenge and deliver iron to the bacterial membrane (17). In addition to these mechanisms, other pathogenic bacteria can produce proteases that degrade iron-transporter proteins, or hemolysins, which are cytotoxic to erythrocytes and other cell types, promoting the uptake of the heme group (18).

*Piscirickettsia salmonis* is the etiological agent of Piscirickettsiosis, a disease that causes high mortalities in the aquaculture industry. This bacterium generates a systemic infection characterized by colonization in several organs, including the kidney, liver, spleen, intestine, ovary, gills, and brain (19). In the brain of *Oncorhynchus kisutch*, the infective dose of *P. salmonis* is 100 times higher than that of the liver or kidney, indicating that this tissue might be a preferred replication site for this bacterium (20). Recent *P. salmonis* genome sequencing and annotation has allowed for identifying a set of orthologous genes involved in iron uptake, indicating that this bacterium can obtain iron from different sources, including ferric iron, heme iron, and free Fe^2+^ (21–25).

Piscirickettsiosis was initially reported as a disease in salmonids (26); however, evidence of this disease exists in other non-salmonid species, such as *Dicentrarchus labrax* (27), *Atractoscion nobilis* (28), *Oreochromis mossambicus*, and *Sarotherodon melanotheron* (29). Furthermore, genomic material (i.e., DNA) of this microorganism has been detected in fish endemic to Chile, including *Eleginops maclovinus, Odontesthes regia, Sebastes capensis*, and *Salilota australis* (30). The role that these native fish could play in disease transmission, as well as the effects that this bacterium could have on the tissues of these endemic organisms, is unknown. Studies by Vargas-Chacoff et al. (31) group reported increased levels antibodies (IgM) in *E. maclovinus* specimens injected with total proteins of *P. salmonis*, with an activation of the intermediate metabolism of the muscle to supply the energetic demand caused by the injection and the high culture density (32). Additionally, Martínez et al. (33) indicated that the injection of live *P. salmonis* modulates the expression of ferritin-H in liver, spleen and muscle of *E*. *maclovinus*, suggesting the possible activation of an iron-limiting system.

*Eleginops maclovinus* (Cuvier, 1830) is a sub-Antarctic notothenioid of the Eleginopsidae (Osteichthyes) family and Notothenioidei suborder. This fish is considered a related species to the Antarctic notothenioids clade (34) and is one of the most eurythermal and euryhaline representatives of this suborder (35, 36). This species habits area associated with salmonid culture centers, subsisting off of unconsumed pellet feed and salmonid excrements (37). The latter suggests an interaction in the natural environment between native and farmed fish, with the consequent transference of microorganisms that presenting different degrees of pathogenicity and resistance to antibiotics. The objective of this study was to evaluate the temporal modulation of iron metabolism genes in liver and brain of *E. maclovinus* challenge with two live strains of *P. salmonis*: LF-89 as reference strain (ATCC^®^ VR-1361™) and Austral-005 strain as an antibiotic resistant.

## Materials and Methods

### Samples

Assessments were conducted with the same specimens and experimental procedures as used in Martínez et al. (33). Briefly, immature fish of *E. maclovinus* (20 ± 5 g body weight) were transferred to the Calfuco Coastal Laboratory facilities of the Faculty of Science (Universidad Austral de Chile, Valdivia, Chile). Fish were acclimated for 4 weeks to the following conditions, as detailed in Vargas-Chacoff et al. (36): 500 L flow-through tanks; 3.1 kg m^−3^ density; 32 psu seawater (1,085 mOsm kg^−1^); 12:12 h light:dark photoperiod; and 12.0 ± 0.5°C. Fish were fed in proportion to 1% body weight once daily with commercial dry pellets (Skretting Nutrece 100) containing 48% protein, 22% fat, 13.5% carbohydrates, 8% moisture, and 8.5% ash. All experimental protocols complied with guidelines for the use of laboratory animals, as established by the Chilean National Commission for Scientific and Technological Research (CONICYT, Spanish acronym) and the Universidad Austral de Chile.

### *P. salmonis* LF-89 and Austral-005 Strains

Inoculates of *P. salmonis* were donated by the Metabolism and Biotechnology Laboratory (Institute of Biochemistry and Microbiology, Faculty of Sciences, Universidad Austral de Chile). LF-89 (ATCC^®^ VR-1361™) was used as a reference strain (38), and Austral-005 strain was used as an antibiotic-resistant representative (39).

### *P. salmonis* LF-89 and Austral-005 Strains Infection Assays

After acclimation, the fish were randomly distributed among rectangular tanks (100 L) and submitted in the experimental treatments: control (fish injected with only the culture medium); LF-89 (fish injected with LF-89 strain); and Austral-005 (fish injected with Austral-005 strain). Each treatment was performed in duplicate. The fish (*n* = 126) were injected with 100 µL of culture medium (control) or with 100 µL at a concentration of 1 × 10^8^ of live bacteria, and sampled at day 1, 3, 7, 14, 21, 28, and 35 postinjection (dpi). Fish were fasted for 24 h before each sampling, netted and submitted to lethal doses of 2-phenoxyethanol (1 mL L^−1^ water). Over the course of the experiment, fish were maintained following Vargas-Chacoff et al. (36): 3.1 kg m^−3^ density, flow-through system, natural photoperiod (12:12 h light:dark), and temperature (12.0 ± 0.5°C). Fish were fed in proportion to 1% body weight once daily with commercial dry pellets (Skretting Nutrece Defense 100) containing 48% protein, 22% fat, 13.5% carbohydrates, 8% moisture, and 8.5% ash.

### Total RNA Extraction

Liver and brain tissues from *E. maclovinus* at different experimental conditions were aseptically extracted and used for total RNA extraction. RNA was extracted from each tissue (50 mg) by homogenization in TRIzol (Ambion) following the manufacturer’s instructions. The RNA pellets were dissolved in diethyl pyrocarbonate water and stored at −80°C. Subsequently, the RNA was quantified at 260 nm on a NanoDrop spectrophotometer (NanoDrop Technologies^®^), and their quality determined by electrophoresis on 1% agarose gel. Total RNA (2 µg) was used as a reverse transcription template to synthesize cDNA, applying MMLV-RT reverse transcriptase (Promega) and the oligo-dT primer (Invitrogen) according to standard procedures.

### RT-qPCR Analysis of Gene Expressions

Reactions were carried out on an AriaMx Real-time PCR System (Agilent). cDNA was diluted to 100 ng and used as a RT-qPCR template with reactive Brilliant SYBRGreen qPCR (Stratagene). Primers were designed for ferritin-H, ferritin-M, ferritin-L, ceruloplasmin, transferrin, hepcidin, haptoglobin, hemopexin, and 18s. Reactions were performed, in triplicate, in a total volume of 14 µL, which contained 6 µL SYBRGreen, 2 µL cDNA (100 ng), 1.08 µL of primers mix, and 4.92 µL of PCR-grade water. The applied PCR program was as follows: 95°C for 10 min, followed by 40 cycles at 90°C for 10 s, 60°C for 15 s, and 72°C for 15 s. Melting curve analysis of the amplified products was performed after each PCR to confirm that only one PCR product was amplified and detected. Expression levels were analyzed using the comparative Ct method (2^−ΔΔCT^) (40). The data are presented as the fold change in gene expression normalized to an endogenous reference gene and relative to the uninfected fish (control). The primers used are listed in Table 1.

**Table 1:** Primer sequences for nutritional immunity used in the experiments.

Primer	Nucleotide sequences (5′ → 3′)	PCR product size	Efficiency liver (%)	Efficiency brain (%)
Ferritin-H Fw	AGTGGAGGCCCTTGAATGTGC	130	101.8	97.3
Ferritin-H Rv	GTCCAGGTAGTGAGTCTCGATGAA			
Ceruloplasmin Fw	GTTTCCAGCCACCTTTCAGACAGT	104	101.8	102.1
Ceruloplasmin Rv	TCGCCTCCATGCCACCTTTAAT			
Transferrin Fw	AACATCCCCATGGGTCTAATCT	124	105.4	100.5
Transferrin Rv	CACTTCCAGCACACTTTGAACA			
Hepcidin Fw	CCGTATACAAGAGCAAGGCG	100	103.2	96.3
Hepcidin Rv	ATCCGAATGCCTTTGTACAGC			
Haptoglobin Fw	ACTGAGCTAACACCAGCTGTA	137	103.4	98.5
Haptoglobin Rv	CCTGCAGCGTAGATGTCTCCA			
Hemopexin Fw	TGATGCAGCAGTAGACGATCCTT	140	103.4	97.4
Hemopexin Rv	GCTGAGCTGGACCATGAAAGCC			
Ferritin-M Fw	CCCGGCTTCGCTCACTTCTTCAA	107	105.9	101.4
Ferritin-M Rv	TCCTGCAGGAAGATGCGTCCTC			
Ferritin-L Fw	AAGCTGCTGGAATATCAGAACAT	123	103.3	102.4
Ferritin-L Rv	CTTCTGGTAGTCCAGGGAAAA			
Housekeeping (18s) Fw	GTCCGGGAAACCAAAGTC	116	104.9	104.9
Housekeeping (18s) Rv	TTGAGTCAAATTAAGCCGCA			

The PCR products were resolved on 2% agarose gel, purified using the E.Z.N.A Gel Extraction Kit (Omega Biotek), and sequenced by Macrogen Inc. Sequences were identified through BLAST analysis (http://blast.ncbi.nlm.nih.gov) against sequences in the NCBI GenBank database. All data are given in terms of relative expression and are expressed as the mean ± standard error of the mean (SEM). PCR efficiencies were determined by linear regression analysis of sample data using LinRegPCR (41).

### Statistical Analyses

Assumptions of variance normality and homogeneity were tested. Data were logarithmically transformed when needed to fulfill conditions for parametric analysis of variance (ANOVA). Gene expression was tested by two-way ANOVA, using the different injections and time as variance factors. ANOVA were followed by a Tukey’s *post hoc* test to identify different groups. Differences were considered significant when *P* < 0.05.

## Results

### Primary Structure of Iron-Related Immune Genes in *E. maclovinus*

Ferritin-M, transferrin, ceruloplasmin, haptoglobin, and hemopexin cDNA fragments were obtained by conventional PCR using *E. maclovinus* liver cDNA as a template and heterologous primers designed from the sequences of other fish species. The PCR products of each gene were purified from 2% agarose gel using the E.Z.N.A Gel Extraction Kit (Omega Biotek) and were sequenced by Macrogen Inc. Sequences were identified through BLAST analysis (http://blast.ncbi.nlm.nih.gov) against the NCBI GenBank. Subsequently, RT-qPCR primers were designed from specific *E. maclovinus* sequences. Partial cDNA coding sequences were obtained and deposited in GenBank under the following accession numbers: MF741822 for ferritin-M, MF741819 for transferrin, MF741824 for ceruloplasmin, MF741821 for haptoglobin, and MF741820 for hemopexin. Ferritin-L was amplified using heterologous primers, and ferritin-H (MF741823) was amplified using published primers (33). The hepcidin sequence was obtained from GenBank under accession number EU221592.

### Mortality after Infection with *P. salmonis*

During the experimental period no mortality or changes in behavior were observed.

### Expression of Iron-Related Immune Genes

**Figure 1:**
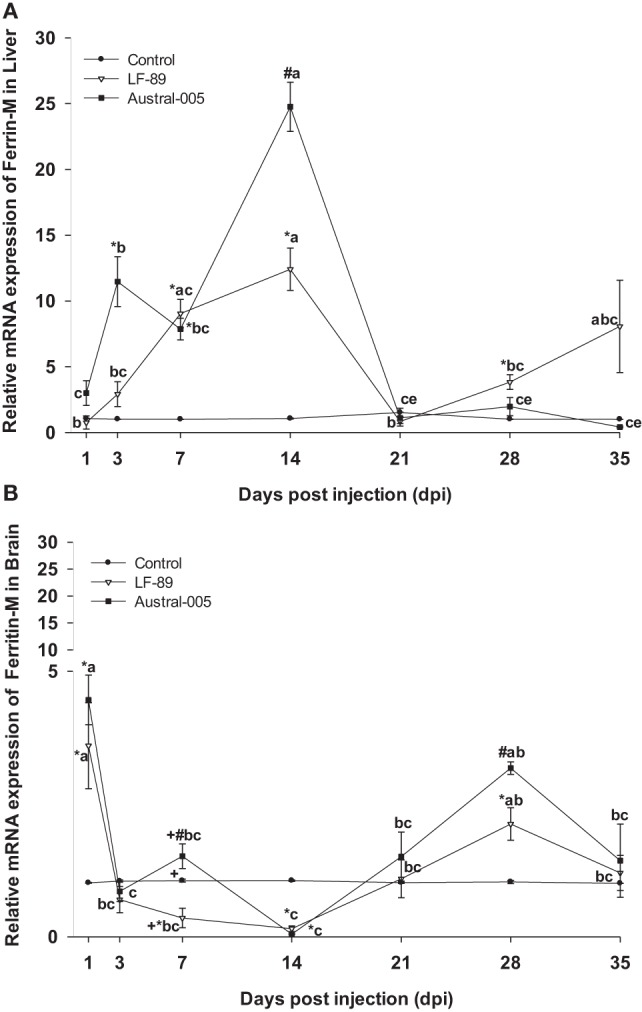
Gene expression of ferritin-M in liver **(A)** and brain **(B)** of *Eleginops maclovinus* specimens injected with *Piscirickettsia salmonis* strain LF-89 (▿) or Austral-005 (^■^) during 35 days. Relative expression was calculated by the 2^−ΔΔCT^ method using the 18s ribosomal protein as the internal reference gene. Each value is the mean ± SEM (*n* = 6). Different letters indicate statistical differences within the same treatment over time. Symbols indicate statistical differences between different treatments (control, LF-89, or Austral-005) at the same sampling time-point [two-way analysis of variance, *P* < 0.05].

Real-time PCR analyses were used to measure the expression of iron-related immune genes in *E. maclovinus* liver and brain tissues at 1, 3, 7, 14, 21, 28, and 35 dpi with *P. salmonis*. Hepatic ferritin-M expression (Figure 1A) significantly increased (5- to 25-fold) at 3, 7, and 14 dpi, with differences in the degree of fold-increase between the LF-89 and Austral-005 groups. In the brain, both strains induced increased ferritin-M expression at 1 and 28 dpi, with statistically significant downregulation at 14 dpi, when compared with the control (Figure 1B).

**Figure 2:**
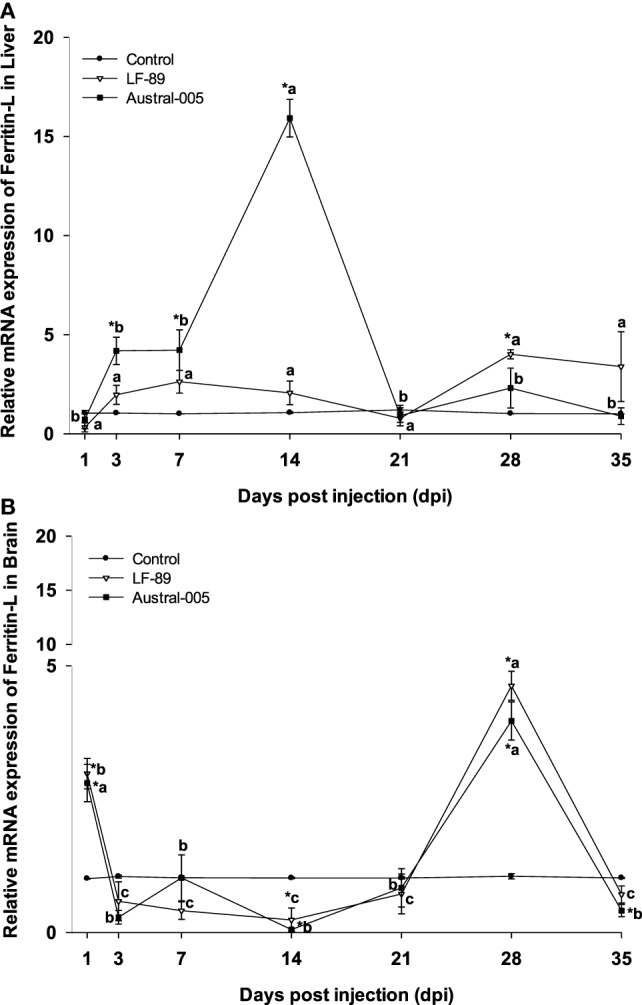
Gene expression of ferritin-L in liver **(A)** and brain **(B)** of *Eleginops maclovinus* specimens injected with *Piscirickettsia salmonis* strain LF-89 (▿) or Austral-005 (^■^) during 35 days. Relative expression was calculated by the 2^−ΔΔCT^ method using the 18s ribosomal protein as the internal reference gene. Each value is the mean ± SEM (*n* = 6). Different letters indicate statistical differences within the same treatment over time. Symbols indicate statistical differences between different treatments (control, LF-89, or Austral-005) at the same sampling time-point [two-way analysis of variance, *P* < 0.05].

Hepatic ferritin-L expression increased in the Austral-005 group at 3, 7, and 14 dpi. Injection with LF-89 only positively modulated hepatic ferritin-L expression at 28 dpi, with no statistical differences when compared with the control during the other sampling time-points (Figure 2A). In contrast to liver expression, ferritin-L was upregulated in the brain at 1 and 28 dpi but downregulated at 14 dpi for both experimental groups (Figure 2B).

**Figure 3:**
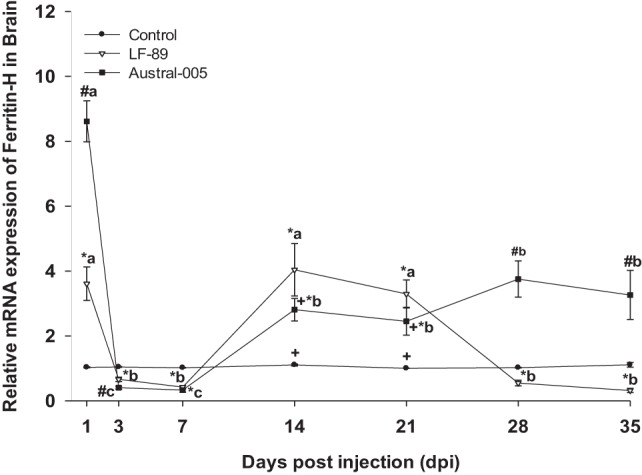
Gene expression of ferritin-H in brain of *Eleginops maclovinus* specimens injected with *Piscirickettsia salmonis* strain LF-89 (▿) or Austral-005 (^■^) during 35 days. Relative expression was calculated by the 2^−ΔΔCT^ method using the 18s ribosomal protein as the internal reference gene. Each value is the mean ± SEM (*n* = 6). Different letters indicate statistical differences within the same treatment over time. Symbols indicate statistical differences between different treatments (control, LF-89, or Austral-005) at the same sampling time-point [two-way analysis of variance, *P* < 0.05].

The expression of ferritin-H in the liver of *E. maclovinus* during *P. salmonis* infection was previously reported elsewhere (33). Specifically, hepatic ferritin-H was upregulated at 3 and 7 dpi, downregulated at 14 dpi, and then upregulated at 21, 28, and 35 dpi by both strains. In the brain, both strains led to significantly increased ferritin-H expression (3.5- to 8.5-fold) at 1 dpi, but drastically decreased expression at 3 and 7 dpi. Following this time-point, specimens injected with Austral-005 showed a steady increase in brain expression from 14 to 35 dpi. In turn, fish injected with LF-89 showed increased ferritin-H expression at 14 and 21 dpi, but brain expression again decreased at 28 and 35 dpi (Figure 3).

**Figure 4:**
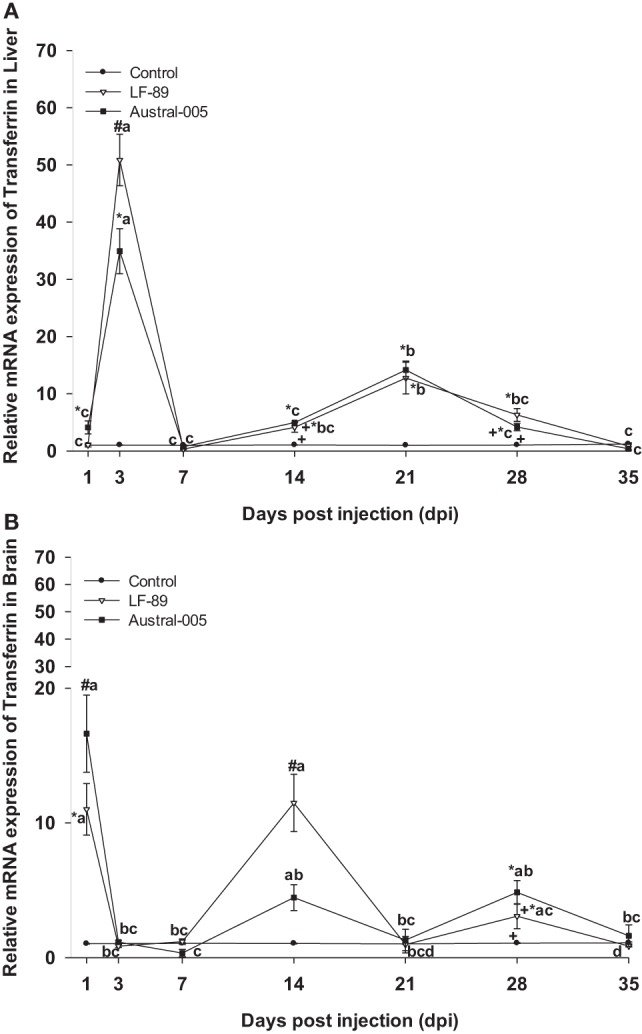
Gene expression of transferrin in liver **(A)** and brain **(B)** of *Eleginops maclovinus* specimens injected with *Piscirickettsia salmonis* strain LF-89 (▿) or Austral-005 (^■^) during 35 days. Relative expression was calculated by the 2^−ΔΔCT^ method using the 18s ribosomal protein as the internal reference gene. Each value is the mean ± SEM (*n* = 6). Different letters indicate statistical differences within the same treatment over time. Symbols indicate statistical differences between different treatments (control, LF-89, or Austral-005) at the same sampling time-point [two-way analysis of variance, *P* < 0.05].

Transferrin expression was similar in both the LF-89 and Austral-005 groups. Expression peaks of hepatic transferrin were recorded at 3 dpi (30- to 50-fold) and 21 dpi (10-fold) (Figure 4A). In the brain, both *P. salmonis* strains modulated transferrin expression, with peaks recorded at 1, 14, and 28 dpi when compared with the control (Figure 4B).

**Figure 5:**
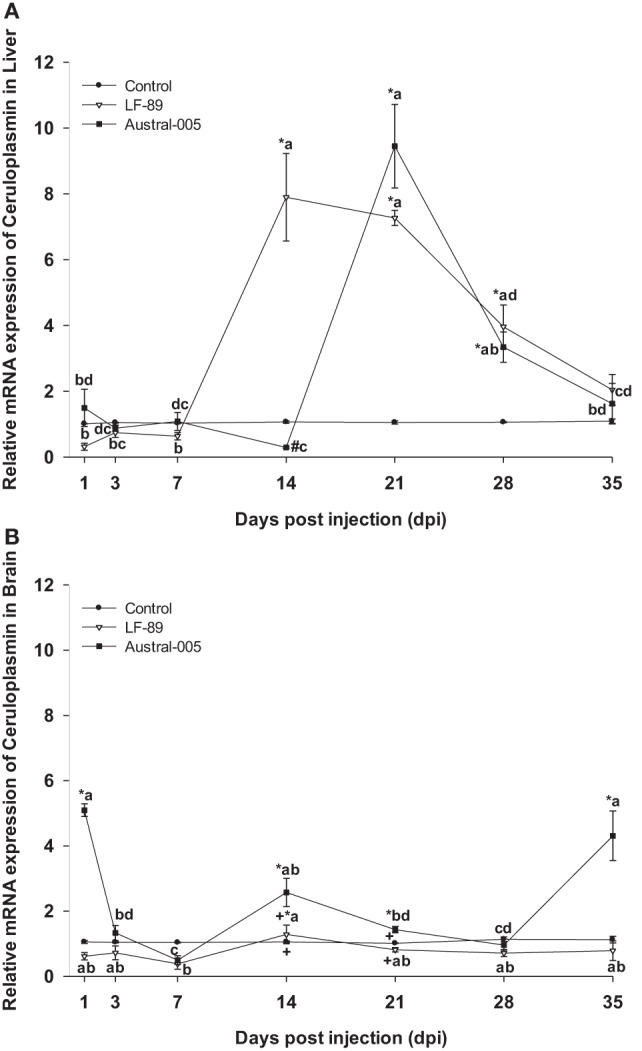
Gene expression of ceruloplasmin in liver **(A)** and brain **(B)** of *Eleginops maclovinus* specimens injected with *Piscirickettsia salmonis* strain LF-89 (▿) or Austral-005 (^■^) during 35 days. Relative expression was calculated by the 2^−ΔΔCT^ method using the 18s ribosomal protein as the internal reference gene. Each value is the mean ± SEM (*n* = 6). Different letters indicate statistical differences within the same treatment over time. Symbols indicate statistical differences between different treatments (control, LF-89, or Austral-005) at the same sampling time-point [two-way analysis of variance, *P* < 0.05].

Ceruloplasmin was similarly expressed in the liver of both experimental groups, with no differences when compared with the control condition at 1, 3, or 7 dpi. While that the LF-89 group showed increased hepatic ceruloplasmin expression at 14 dpi, the Austral-005 group presented increased expression at 21 dpi (Figure 5A). In the brain, only the Austral-005 strain evidenced upregulated ceruloplasmin expression (1, 14, 21, and 35 dpi). The LF-89 group, however, showed no statistical differences when compared with the control during the experiment in brain (Figure 5B).

**Figure 6:**
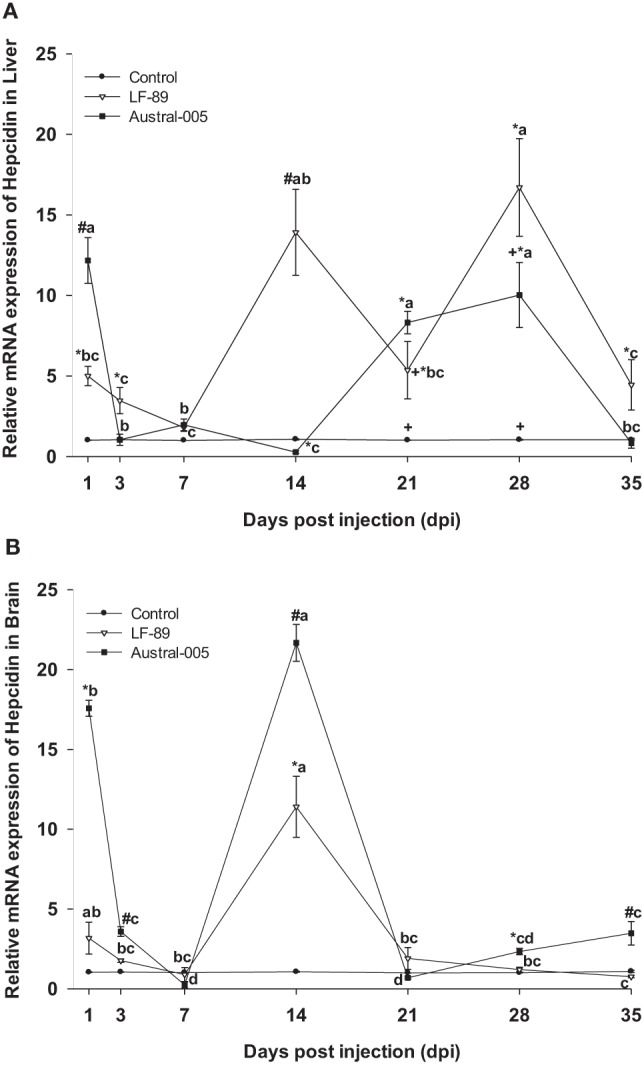
Gene expression of hepcidin in liver **(A)** and brain **(B)** of *Eleginops maclovinus* specimens injected with *Piscirickettsia salmonis* strain LF-89 (▿) or Austral-005 (^■^) during 35 days. Relative expression was calculated by the 2^−ΔΔCT^ method using the 18s ribosomal protein as the internal reference gene. Each value is the mean ± SEM (*n* = 6). Different letters indicate statistical differences within the same treatment over time. Symbols indicate statistical differences between different treatments (control, LF-89, or Austral-005) at the same sampling time-point [two-way analysis of variance, *P* < 0.05].

The hepatic expression profile of hepcidin was dependent on the injected bacterial strain. Gene expression was upregulated 1, 14, 21, 28, and 35 dpi in the LF-89 group, whereas hepcidin was upregulated in the Austral-005 group at 1, 21, and 28 dpi. Furthermore, the Austral-005 group presented negative modulation of hepatic hepcidin expression at 14 dpi, when compared with the control group (Figure 6A). In the brain, hepcidin was also differentially modulated over time in the Austral-005 group, with significant peak expressions recorded at 1, 3, 14, 28, and 35 dpi. By contrast, the LF-89 group only showed significant brain hepcidin modulation at 14 dpi (Figure 6B).

**Figure 7:**
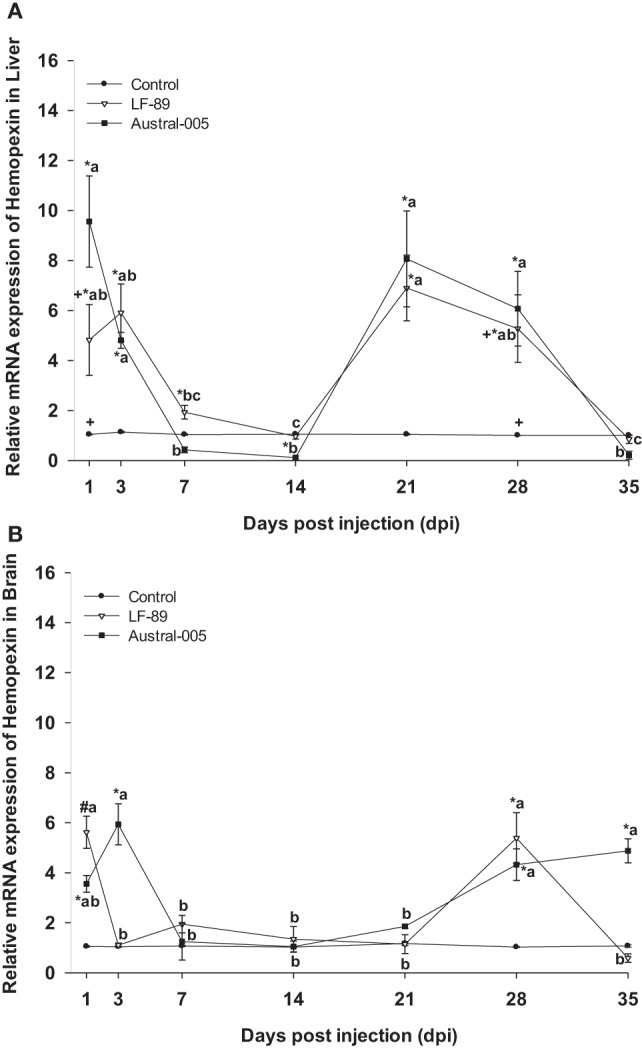
Gene expression of hemopexin in liver **(A)** and brain **(B)** of *Eleginops maclovinus* specimens injected with *Piscirickettsia salmonis* strain LF-89 (▿) or Austral-005 (^■^) during 35 days. Relative expression was calculated by the 2^−ΔΔCT^ method using the 18s ribosomal protein as the internal reference gene. Each value is the mean ± SEM (*n* = 6). Different letters indicate statistical differences within the same treatment over time. Symbols indicate statistical differences between different treatments (control, LF-89, or Austral-005) at the same sampling time-point [two-way analysis of variance, *P* < 0.05].

For both the LF-89 and Austral-005 groups, liver expression of hemopexin was significantly upregulated from 1 to 3 dpi before decreasing at 7 and 14 dpi, increasing at 21 and 28 dpi, and finally decreasing to basal levels at 35 dpi (Figure 7A). In the brain, hemopexin expression in the LF-89 group only significantly increased, when compared with the control, at 1 and 29 dpi. For the Austral-005 group, brain expression of hemopexin was increased at 1, 3, 28, and 35 dpi but presented no differences when compared with the control at 7, 14, and 21 dpi (Figure 7B).

**Figure 8:**
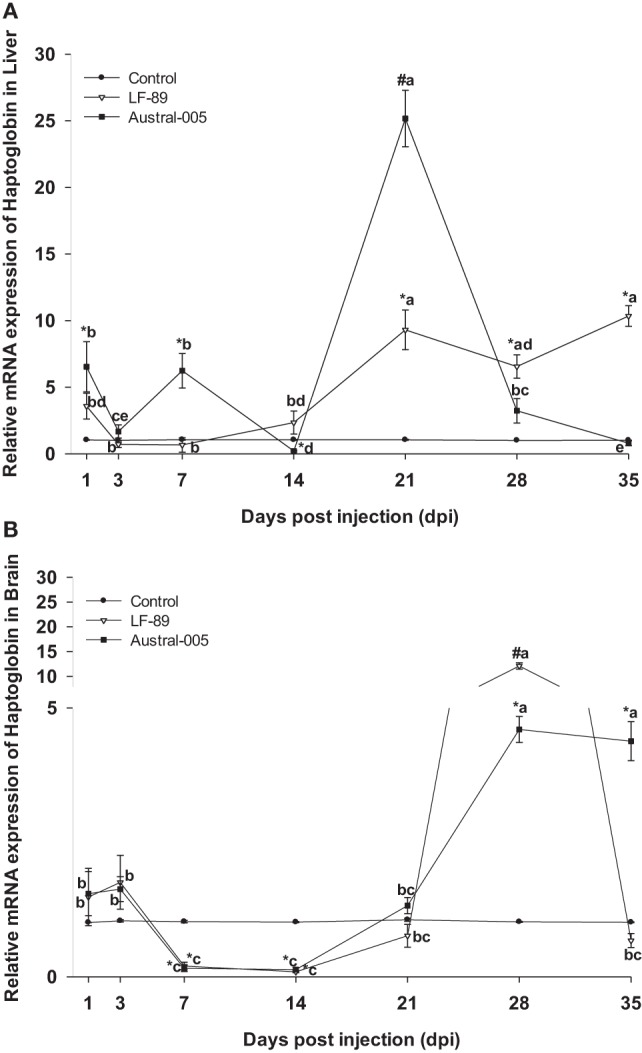
Gene expression of haptoglobin in liver **(A)** and brain **(B)** of *Eleginops maclovinus* specimens injected with *Piscirickettsia salmonis* strain LF-89 (▿) or Austral-005 (^•^) during 35 days. Relative expression was calculated by the 2^−ΔΔCT^ method using the 18s ribosomal protein as the internal reference gene. Each value is the mean ± SEM (*n* = 6). Different letters indicate statistical differences within the same treatment over time. Symbols indicate statistical differences between different treatments (control, LF-89, or Austral-005) at the same sampling time-point [two-way analysis of variance, *P* < 0.05].

Finally, haptoglobin in the liver increased in expression at 21 dpi (10- to 25-fold) for both the LF-89 and Austral-005 groups. Slight increases in expression (sixfold) were observed during the first days post-injection for only the Austral-005 group. In contrast, the LF-89 group presented increased expression at 1 dpi but no differences when compared with the control at 3, 7, and 14 dpi. Hepatic haptoglobin expression in the LF-89 group then steadily increased from 21 to 35 dpi (Figure 8A). In the brain, haptoglobin presented no variations in expression for both *P. salmonis* strains during the first days postinjection. However, this gene was then downregulated in the LF-89 and Austral-005 groups, when compared with the control, at 7 and 14 dpi. At 28 and 35 dpi, the Austral-005 group showed drastically increased haptoglobin expression in the brain, whereas the LF-89 group only showed such an increase at 28 dpi (Figure 8B).

## Discussion

The competition for iron between pathogens and hosts underscores the need to evaluate how iron-related immune genes modulate expression to withhold this nutrient and, consequently, reduce bacterial load (42). In the present study, the partial coding sequences (cDNA) of proteins implicated in *E. maclovinus* iron metabolism were identified, including ferritin-M, transferrin, ceruloplasmin, hemopexin, and haptoglobin. These genes, together with ferritin-H, ferritin-L, and hepcidin were modulated in response to the injection with *P. salmonis*. The expression profiles of the transferrin and hemopexin genes in the liver, as well as the expression profiles of ferritin-M, ferritin-L, and transferrin in the brain, were similar for both experimental groups (i.e., injected with LF-89 or Austral-005 strains). Nevertheless, the remaining genes presented tissue-specific expression profiles that varied in relation to the injected bacterial strain and sampling time-point. It is probable that genetic differences between the bacterial strains would induce the host to modulate certain genes much more than others, both in the liver and in the brain. The Austral-005 strain is antibiotic resistant maintained in the AUSTRAL-SRS medium (39, 43). In turn, LF-89 is the only reference strain originally obtained from *O. kisutch* (38) and it is used worldwide.

The liver regulates iron homeostasis through the synthesis and storage of proteins involved in iron metabolism (44). In the present study, the ferritin-H, ferritin-M, and ferritin-L genes responded to bacterial infection, through a modulation in its expression during the experimental course, with tissue-specific expression profiles. The increased expression of these genes, in the liver and brain, could be consistent with a decrease in serum iron content and the need to increase iron storage so as to limit availability for bacterial growth. This would be in line with existing literature, where the expression of ferritin is downregulated under conditions of iron deficiency and upregulated during iron abundance in *D. labrax* (45). Other studies indicate that in conditions of inflammation and infestation, the expression of iron-related immune genes is upregulated (46–49). Additionally, the synthesis of proteins involved in cellular iron uptake and storage is modulated by cellular iron levels. Under conditions of iron deficiency, iron regulatory proteins actively bind to iron responsive elements and stabilize TfR mRNA, while also decreasing the translation of ferritin mRNA. Conversely, high iron levels decrease iron response element binding activity, leading to efficient ferritin mRNA translation and decreased TfR mRNA stability, thus favoring iron uptake (50).

The hepatic expression profiles for ferritin-M and ferritin-L were similar, increasing during the first 2 weeks of the challenge, particularly in fish injected with the Austral-005 strain. A similar profile expression was reported for ferritin-L subunit in *Salmo salar* (23) and ferritin-H en *E. maclovinus* (33), both challenged with *P. salmonis*. In the brain, iron might be undergoing rapid storage at 1 dpi and release at 14 dpi, as per the results obtained for ferritin-M and -L. However, the expression of both ferritins increased again at 28 dpi. The expression of ferritin-H in the brain also increased at 1 dpi, fell at 3 and 7 dpi, and finally became generally upregulated during the remainder of the experimental period. It is probable that the different ferritin (H, M, and L) expression patterns are due to the different iron needs of each tissue, as well as the necessity to synthesize one subunit over another as per functional variations. High ferroxidase activity is presented by H-rich ferritins, resulting in more active iron oxidation and sequestration, as well as more pronounced antioxidant functions (51). In turn, L-rich ferritins form more physically stable molecules that may contain a greater amount of iron in the cavity and may have more pronounced iron-storage functions (51). Finally, M-rich ferritins present both the ferroxidase function of ferritin-H and the storage function of ferritin-L (10).

In the present study, the upregulated expression of ferritins (H, M, and L) coincided in some cases with an increased expression of the transferrin gene in the liver and brain. This would indicate that transferrin might be rapidly removing circulating iron through reversible binding with this element. Another mechanism has been reported for mammals, where iron uptake can be independent of transferrin and involve the action of a ferritin secretor able to deliver iron to multiple organs, including the brain. Furthermore, iron uptake is greater when iron is delivered by ferritin-H when compared with ferritin-L (52). On the other hand, the transferrin gene expression in the brain of teleost fish is species-specific, detecting in liver, kidney, and stomach of *Salmo salar*, but not in brain (53), compared to *Gadus morhua*, a species in which transferrin is synthesized in the brain (54). These results shown the first instance of transferrin mRNA detection in a fish of Antarctic origin (Notothenioidei), suggesting that local synthesis of this protein could play a role in the immune response of *E. maclovinus*.

Ceruloplasmin, a homologous protein to intestinal hephaestin (7) can be synthetized in the liver (55) and in low amounts spleen, brain, gills, intestine, muscle, skin, and stomach of *Ictalurus punctatus* (56). In the present study, ceruloplasmin expression levels in the liver were greater than those found in the brain. Furthermore, ceruloplasmin brain expression was unchanged, when compared with the control, following injection with the LF-89 strain, and Austral-005 only incremented gene transcription at 1, 14, and 35 dpi. It is possible that genetic differences between the bacterial strains would result in a differential response in brain tissue. Prior studies assessing the modulation of ceruloplasmin in the brain of fish do not exist, but in mammals, cells of the central nervous system synthesize ceruloplasmin as a glycophosphatidylinositol-anchored protein (57). This phenomenon suggests that this isoform interacts with ferroportin to transfer iron from cells to the blood, i.e., cross the blood–brain barrier (57). In the liver, ceruloplasmin expression was upregulated from 14 to 28 dpi in the LF-89 group and from 21 to 28 dpi in the Austral-005 group. These expression patterns indicate that ceruloplasmin acts as a positive acute-phase protein able to promote the uptake of transferrin iron and subsequent transport toward bone marrow or other precise tissue. Such as function would be in line with findings published for *I. punctatus*, a species that increases the gene expression of ceruloplasmin in the liver after injection with *Edwardsiella ictaluri* (56). In Antarctic notothenioids, ceruloplasmin has been found in the liver and head kidney, thereby suggesting that increased ceruloplasmin expression could prevent the deleterious accumulation of ferrous iron in tissues (58).

In mammals, bonding between hepcidin and ferroportin induces the internalization and posterior degradation of ferroportin, meaning decreased iron export (11). In the currently conducted analyses, the genic expression levels of hepcidin were variable in both the liver and brain, being the expression in liver increased 20-fold higher than the control condition, with elevated expression maintained during nearly the entire experimental period for the LF-89 group and on determined days for the Austral-005 group. In the brain, genic expression of hepcidin differed from that in the liver. In particular, Austral-005 injection resulted in increased gene expression during nearly the entire experimental period, whereas LF-89 injection resulted in increased expression only at 14 dpi. It is possible that the tissue-specific expression profiles obtained for this gene would be due not only to the physiological functions of hepcidin in regulating iron homeostasis (59, 60) but also to inherent antimicrobial properties (61). Ganz and Nemeth (62) indicate that the antibacterial properties of hepcidin could be a result of participating in plasma iron depletion, as corroborated by various studies supporting that genic hepcidin expression is upregulated under inflammatory conditions (63–67). In Antarctic notothenioid fish exists a type of hepcidin composed by four, not eight, cysteine residues, suggesting an adaptive evolution of this gene to a cold climate (68).

Proinflammatory cytokines, such as IL-6, stimulate the transcriptional upregulation of hepcidin, triggering, and enhancing the hypoferric response of inflammation (6). However, prolonged iron uptake could limit the availability of this nutrient to erythroid precursors, which use iron in the heme group (69). This group forms a part of hemoproteins, including myoglobin and hemoglobin. The last belongs to a family of positive acute-phase proteins, whose synthesis is induced by inflammatory cytokines (70–72). In the present study, the genic expression of haptoglobin varied over the course of the experimental period for both analyzed tissues. Hepatic gene expression increased in transcription during the first days postinjection and at 21 dpi of fish injected with the Austral-005 strain. However, fish injected with LF-89 showed increased expression of this gene during the final days of the challenge period. The haptoglobin expression profile in the brain differed from that found in the liver. Particularly, downregulated gene transcription was recorded for both strains at 7 and 14 dpi. At 28 and 35 dpi, the Austral-005 group evidenced increased transcription, whereas the LF-89 group only showed increased transcription at 28 dpi. These results suggest that this gene, as with the other evaluated genes, undergoes tissue-specific up- and downregulation following infection with *P. salmonis*. This positive acute-phase protein might also play a fundamental role in the immune response of *E. maclovinus*, as has been reported in a prior study regarding the upregulation of haptoglobin and serum amyloid A in the liver following bacterial and/or viral stimulation (73).

Uptake of the haptoglobin–hemoglobin complex in mammals occurs by endocytosis through the CD163 receptor, which is exclusively expressed in monocytes/macrophages (70). Formation of this complex under hemorrhagic, hemolytic, and/or cell-injury conditions prevents the negative effects of iron contained in the hemo group (71). Hemopexin, a protein with increased affinity for the free hemo group prevents the negative effects of iron contained within the heme group (74). One homolog to hemopexin, termed warm-temperature acclimation-related 65 kDa protein (wap65), has been identified in teleost fish (75) and cartilaginous fish (76). In the present study, the expression of hemopexin in the liver and brain was positively modulated during the experimental course, probably due to their participation in the immune response against *P. salmonis*. The present findings are in line with that reported for *Misgurnus mizolepis*, a species in which the wap65-2 isoform was found upregulated in the liver and brain following a challenge with *Edwardsiella tarda* (75).

Compared to *Oryzias latipes* and *I. punctatus*, where the transcripts of wap65-2 are restricted to the liver y wap65-1 transcripts can be found in various tissues (77, 78). Orthologous genes exist for hemolysins and respective secretion components in the *P. salmonis* genome, suggesting that this bacterium could acquire iron from host hemo groups (23).

## Conclusion

This study is the first to provide cDNA sequences for ferritin-M, transferrin, ceruloplasmin, haptoglobin, and hemopexin in the sub-Antarctic notothenioid *E. maclovinus*. The expression of these genes, together with that of ferritin-L and hepcidin, presented transcriptional differences following the challenge with *P. salmonis* LF-89 or Austral-005 strain. Indeed, tissue-specific expression profiles were found dependent on the sampling time-point and injected bacterial strain. These variations in transcript abundances suggest the activation of an innate immune response via iron deprivation, so as to limit bacterial growth. Future studies are needed to complement how nutritional immunity acts in this sub-Antarctic fish at a protein and functional level, as well as to establish differences when compared with mammals, organisms in which nutritional immunity has been widely studied.

## Ethics Statement

All experimental protocols complied with guidelines for the use of laboratory animals, as established by the Chilean National Commission for Scientific and Technological Research (CONICYT, Spanish acronym) and the Universidad Austral de Chile.

## Author Contributions

DM, LV-C, and AY conception and design of research; DM, RO, JP, and LV-C performed the experiments; DM, RO, AR, and JP analyzed the samples; DM, JP, RO, and LV-C analyzed the data; DM, AR, LV-C, and AY interpreted the results of experiments; DM and RO prepared the figures; DM, AR, LV-C, and AY drafted the manuscript; LV-C and DM edited and revised the manuscript; DM and LV-C approved the final version of manuscript.

## Conflict of Interest Statement

The authors declare that the research was conducted in the absence of any commercial or financial relationships that could be construed as a potential conflict of interest.
